# Artificial Intelligence for Perovskite Additive Engineering: From Molecular Screening to Autonomous Discovery

**DOI:** 10.3390/molecules31030440

**Published:** 2026-01-27

**Authors:** Xin-De Wang, Zhi-Rui Chen, Wen-Kao Li, Peng-Jie Guo, Cheng Mu, Ze-Feng Gao, Zhong-Yi Lu

**Affiliations:** 1 School of Physics, Renmin University of China, Beijing 100872, China; 2 School of Chemistry and Life Resource, Renmin University of China, Beijing 100872, China

**Keywords:** perovskite solar cells, additive engineering, machine learning, high-throughput screening, autonomous discovery

## Abstract

Additive engineering plays a crucial role in enhancing the performance of perovskite solar cells (PSCs), yet identifying suitable additives within the vast chemical space remains a significant challenge. This paper describes a paradigm shift in additive discovery from trial-and-error methods to AI-driven approaches. We first establish the physicochemical foundations of additive engineering and the descriptors commonly employed in machine learning algorithms. Next, we discuss intelligent process optimization, highlighting how active learning algorithms effectively tune complex precursor formulations with minimal experimental iterations. Additionally, we explore the role of AI in mechanism elucidation and the potential prospects of generative models in the field of additives. Finally, we emphasize the emerging trend of integrating large language models with autonomous laboratories for closed-loop autonomous discovery, offering a promising pathway to accelerate the commercialization of PSCs.

## 1. Introduction

The Power Conversion Efficiency (PCE) of Perovskite Solar Cells (PSCs) has made an astonishing leap in just two decades, starting at less than 4% and now approaching 27% [1,2,3,4]. Despite such a great breakthrough, the commercialization of PSCs is still greatly hindered, especially in terms of stable operation and large-scale manufacturing [5,6]. Achieving robust devices requires a holistic optimization of the entire device stack, including binary oxide ceramics (e.g., ${\mathrm{TiO}}_{2}$, ZnO, ${\mathrm{SnO}}_{2}$) commonly used as charge transport layers [7]. The ionic nature of the perovskite lattice makes polycrystalline films susceptible to defect formation, and while recent studies indicate that bulk vacancies can mediate ion migration and degradation channels [8], defects at grain boundaries and on surfaces are nevertheless susceptible to act as non-radiative recombination centers that accelerate decomposition in response to temperature, heat, and light [9,10,11,12].

In order to solve the above mentioned problems, additive engineering has become one of the most widely used countermeasures. It is worth noting that this field encompasses diverse strategies. For instance, plasmonic additives have been shown to leverage sophisticated physical phenomena to enhance efficiency [13], a complexity that distinctively underscores the need for deep physical understanding. While acknowledging these broad physical approaches, in this review, we strictly define “additive engineering” as the incorporation of extrinsic molecular agents, specifically including defect passivators, solvent additives, and crystallization modifiers, into the perovskite precursor or at grain boundaries. By introducing functional molecules such as Lewis bases, ammonium salts, and large organic cations into precursors or grain interfaces, researchers have been able to modulate crystallization kinetics and passivate defects [14]. Conversely, topics such as the stoichiometric tuning of bulk perovskite composition (e.g., cation or halide alloying) and the independent engineering of charge transport layers are considered out of the scope of this work. However, the choice of additives currently faces the problem of combinatorial explosion. Although the selection of additives is somewhat constrained by consideration of various practical conditions, such as compatibility with precursor solutions, heat treatment, chemical stability, and so on. However, when considering the usual chemical molecules, the chemical space constituted by potential small molecule additives is still very large [15,16]. In this case, the traditional trial-and-error method is inefficient and laborious in the face of such a large component space, and often only finds local optimal points [17].

In the above context, the introduction of Artificial Intelligence (AI) and Machine Learning (ML) methods is driving a paradigm shift in materials discovery from raw data analysis to active rational design [18,19,20]. In the field of molecular screening, ML modeling with molecular descriptors has made it possible to discover suitable additives from large chemical libraries [21]. For example, the recently developed Co-PAS (Co-Pilot for Perovskite Additive Screener) framework combines molecular backbone classification with deep learning for screening effective organic additives, thus overcoming the prediction bias common in small datasets [22]. In addition to static screening, Active Learning (AL) algorithms such as PLATIPUS and Bayesian Optimization (BO) have demonstrated the ability to optimize complex formulations and process windows with limited experimental budgets. These methods have significantly accelerated the discovery of crystal phases and efficient devices by intelligently balancing exploration and exploitation, and they have achieved better results with fewer iterations compared to random sampling [16,23].

In addition, large language models (LLMs) have recently begun to play a role in the perovskite field. Unlike traditional machine learning models that rely on structured tabular data, domain-specific large language models such as Perovskite-R1 have the unique ability to mine specialized knowledge from the scientific literature and autonomously select appropriate additives based on learned chemistry [24,25]. And these LLMs have the potential to enable a closed-loop discovery process from recipe generation to automated synthesis and characterization when used in conjunction with robotic platforms like PASCAL (Perovskite Automated Spin Coat Assembly Line), which paves the way for future automated laboratories that could potentially operate 24/7 without human intervention [26].

The purpose of this review is to provide an overview of AI-driven PSC additive engineering in general, with a particular focus on advances from discriminative screening to generative design. We begin by describing the physicochemical foundations of additive engineering and discuss some of the relevant AI foundations for additive screening [27]. We then examine process optimization strategies, highlighting how AL responds to complex trade-offs in precursor composition and processing conditions [16,23]. We also discuss the role of interpretable AI, such as SHAP (Shapley Additive Explanations) analysis, in revealing defect passivation mechanisms, effectively opening the black box of ML models [28,29]. Finally, we outline emerging directions such as the application of LLMs in perovskites and look at future directions towards fully automated materials discovery [19].

## 2. Physicochemical Basis of Additive Engineering

### 2.1. Defect Passivation Mechanisms

The properties and stability of polycrystalline perovskite films are fundamentally limited by the high defect density located at grain boundaries and interfaces within the multilayer device stack. It is crucial to recognize that within the multilayer device structure, these defects are particularly detrimental at the interfaces between the perovskite and charge transport layers (electron or hole transport layers), where they severely impede carrier extraction. Common defects in polycrystalline perovskite films include under-coordinated ${\mathrm{Pb}}^{2+}$ sites and halogen vacancies, which may be positively charged, while A-site vacancies are usually negatively charged. These charged defects often act as non-radiative complex centers or ion migration channels under certain conditions, and thus, these defect sites not only reduce the open-circuit voltage and fill factor, but also act as permeation channels for moisture and oxygen, accelerating the degradation of the device [30]. Therefore, the main goal of additive engineering is to neutralize these defects through specific chemical reactions with the help of additive molecules [14,31].

The most widely used strategy involves Lewis acid–base interactions. Since under-coordinated ${\mathrm{Pb}}^{2+}$ ions act as Lewis acids, effective additives usually have Lewis base functional groups, typically such as amino (${\mathrm{NH}}_{2}$), carboxyl (COOH), carbonyl (C=O), or thiol (SH) parts. According to the hard-soft acid–base (HSAB) principle, the binding affinity is maximized when the electronic softness of the donor group matches the ${\mathrm{Pb}}^{2+}$ site [32]. These groups can donate lone pairs of electrons to under-coordinated ${\mathrm{Pb}}^{2+}$ sites to form Lewis adducts, which can help to passivate the dangling bonds and reduce the density of deep energy level traps or non-radiative complex centers. This coordination effect is essential to minimize non-radiative complex losses and is a fundamental logical basis for molecular screening.

At the same time, weak molecular interactions play a key role in stabilizing the perovskite lattice and inhibiting ion migration. Additives containing hydrogen bond donors, such as primary or secondary ammonium molecules, contribute to Lewis acid–base interactions with the perovskite framework. These interactions modulate crystallization kinetics by stabilizing the intermediate phase and retarding nucleation, while blunting low-coordination surface defects [33]. Meanwhile, large cations acting through strong ionic coupling, such as quaternary ammonium salts, have been shown to act as hydrophobic spacers, enhancing phase stability and moisture resistance [21,34]. Together, these mechanisms contribute to the enhanced stability observed in various perovskite devices.

Understanding these physicochemical mechanisms provides the necessary theoretical foundation for feature engineering in ML models. As summarized in Table 1, specific molecular descriptors, such as the number of hydrogen bond donors, Topological Polar Surface Area (TPSA), and MACCS bonds, are used as input features precisely because they serve as quantitative proxies for the ability of the molecule to perform these passivating behaviors [21,22,27]. By mapping chemical structure and functional group information into numerical descriptors or fingerprints, ML models can be used as efficient coarse screening or prioritization tools for unseen candidate molecules. These models can significantly reduce the search space before applying more computationally expensive computational simulations or experimental validation.

### 2.2. Regulation of Crystallization Kinetics

Crystallization of perovskite thin films is a complex phase-transition process dictated by the competition between nucleation and crystal growth, a process that can be theoretically described by the LaMer model [36]. Defining these underlying physical laws is a prerequisite for constructing valid AI search spaces. Recent mechanistic studies, however, have provided a finer-grained view of additive-engineered thin films. Maschwitz et al. demonstrated that, particularly for typical Lewis-base additives in standard DMF/DMSO solvent systems, these agents may not primarily retard the initial nucleation phase as previously thought, but rather promote post-nucleation grain coarsening by enhancing ionic migration between grain boundaries, which in turn determines the final morphology [37]. However, we emphasize that these conclusions are system-specific. The dominance of coarsening versus nucleation suppression strictly depends on the additive class, such as coordination strength, and the solvent environment. Therefore, regulating the reaction kinetics to balance the nucleation density and promote crystal growth is essential for fabricating high-efficiency devices [38].

Intermediate phase engineering is a highly effective strategy for modulating these kinetics. Lewis base molecules like dimethyl sulfoxide (DMSO) are often introduced as cosolvents or additives to coordinate with lead iodide and form stable intermediate adducts [39]. The crystallization of perovskite thin films is essentially governed by the LaMer model, in which controlling the degree of supersaturation is crucial for high quality formation. The dominant strategy employs mesophase engineering, utilizing Lewis base additives to form adducts with precursors. This interaction increases the nucleation barrier and retards rapid precipitation, resulting in a controlled evolution from the mesophase to polycrystalline films [33,40].

To complement this model of nucleation inhibition, recent studies have emphasized the role of additives in the subsequent annealing stage.Maschwitz et al. showed that additives promote grain coarsening by enhancing ionic mobility between grain boundaries, rather than acting only in the initial nucleation stage [37]. In addition, Li et al. demonstrated that specific chemical interactions, such as hydrogen bonding, modulate the reactivity of the nucleation pre-intermediate, thereby tightly controlling the phase selectivity and dimensionality [17]. These physicochemical mechanisms emphasize the need for ML models to learn from both electronic descriptors (for coordination strength) and structural features (for kinetic mobility). In addition to these overall chemical interactions, recent ML studies have highlighted the critical role of physical conformation in surface interactions. Specifically, Ragni et al. [29] revealed that the structural flexibility of the modified molecule is the decisive factor. Unlike rigid molecules, flexible additives can extend more efficiently at the perovskite interface, thereby maximizing surface coverage and defect passivation efficacy.

These physical and chemical mechanisms inform feature selection in data-driven models. Geometric and solubility descriptors are not just statistical variables, but quantitative proxies for kinetic behavior. For example molecular weight and rotatable bonds encode spatial hindrance and entropy penalties [29], while specific interaction metrics, such as the number of hydrogen bonds, encode phase selectivity and reaction kinetics [17] (see Table 1). By introducing this physicochemical prior knowledge, ML algorithms can learn to distinguish between molecules that disrupt crystal continuity and those that promote the formation of high-quality films.

## 3. AI Infrastructure for Additive Screening

### 3.1. Data Infrastructure and Standardization

High-fidelity data constitutes the bedrock for training robust machine learning models, yet the literature in the perovskite field currently suffers from severe fragmentation and a lack of standardization [3]. Currently, data acquisition in this field still typically relies on manual extraction from literature or direct generation from experiments, which often results in datasets being too small [21]. Several efforts have been made to address this issue. The most prominent contribution in this field is the Perovskite Database Project, which has compiled over 42,000 device entries and stands as the most authoritative open-source repository currently available in this domain [41]. Despite these resources, data heterogeneity remains a critical bottleneck. Preparation conditions across different laboratories, including spin-coating kinetics, annealing humidity, and glove box environments, exhibit significant variations. This introduces substantial spurious noise into the dataset, thereby hindering generalization capabilities of the model [27,41].

For additive engineering, the lack of standardized metadata significantly increases model training complexity and may limit the generalizability of predictions. Critical experimental variables, such as additive purity, incorporation schemes, and precise metering ratios, are often underreported. A prime example is the ambiguity surrounding concentration units. A reported 1% concentration could denote a molar ratio relative to lead, a mass ratio relative to the solvent, or a volume fraction. This ambiguity makes data integration and harmonization exceptionally challenging [41,42].

Moreover, compared to the computer vision field with its million-sample datasets, many additive screening studies in perovskite research still rely on relatively small experimental datasets, typically comprising hundreds to thousands of validated data points. This scarcity of data exacerbates the risk of overfitting. Therefore, scenarios like this require algorithms capable of efficiently utilizing available data, such as Gaussian processes for quantifying uncertainty [16], or transfer learning strategies pre-trained on large chemical databases like ZINC [22].

### 3.2. Molecular Representation and Feature Engineering

To enable machine learning algorithms to process chemical information, discrete molecular structures must be converted into machine-readable numerical vectors. This process, known as feature engineering, bridges the gap between chemical principles and computational science [22,27]. The Simplified Molecular Input Line Entry System (SMILES) is frequently used as the standard input format for these workflows [20]. While SMILES strings enable algorithms to parse structures linearly, they typically require normalization to ensure a one-to-one correspondence between structures and strings [22].

Effective screening typically relies on the identification of specific functional groups that facilitate coordination with perovskite defects [21,22]. Although classical representations like MACCS Keys explicitly encode these structures, recent studies employing the Co-PAS framework demonstrate that supplementing them with advanced encoders can capture richer structural semantics. Furthermore, combining this approach with RDKit-based skeleton extraction enables more precise classification [22]. It is noteworthy that beyond fixed-length vector representations, an increasing number of novel approaches are exploring graph representations, where atoms are treated as graph nodes and chemical bonds as graph edges, to capture global topological features using Graph Neural Networks (GNNs) [19,43]. However, descriptor-based methods remain dominant in small-data scenarios [27].

**Figure 1:**
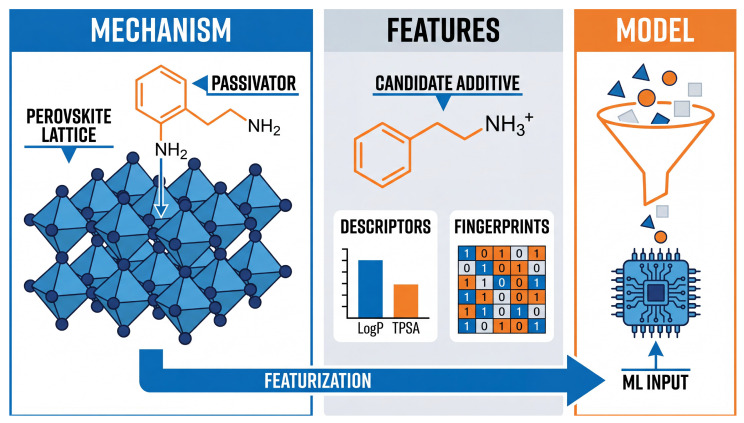
Schematic of the characterization workflow for AI-driven additive screening. The process begins with identifying physical interaction mechanisms, such as the coordination of passivators with perovskite lattice defects (**left**). Candidate molecules are then transformed through characterization into numerical vectors recognizable by machine learning (**center**), including extraction of physicochemical descriptors (e.g., LogP, TPSA) and topological fingerprints (e.g., MACCS keys). These serve as inputs for machine learning models (**right**), effectively bridging chemical structures with predictive algorithms.

Physicochemical descriptors computed via chemoinformatics tools provide critical mechanistic insights. Based on the theoretical framework established in Section 2 (Table 1) and the characterization workflow depicted in Figure 1, high-throughput screening studies predominantly select LogP, TPSA, and hydrogen bond count as input features [21,35]. The model treats these physical quantities not merely as statistical variables, but as quantitative proxies for the hydrophobicity, dissolution kinetics, and defect anchoring efficiency discussed earlier. This enables the algorithm to explicitly learn the structure-property relationships governing passivation and crystallization, while avoiding redundant atomic-scale simulations. Advanced feature importance analysis further refines these insights. For instance, methods like SHAP analyze the specific roles played by atomic properties, such as the electronegativity of chlorine atoms, and molecular flexibility, characterized by rotatable bonds, in maximizing open-circuit voltage and uniform coverage [29].

### 3.3. Machine Learning Algorithms and Screening Frameworks

Given the high-dimensional nature of chemical space and the common data scarcity in additive engineering, selecting an appropriate ML algorithm is crucial. For discriminative tasks, ensemble learning methods such as Random Forests (RF) and Gradient Boosting Decision Trees (GBDTs) are often favored for their balanced performance and efficiency [44,45,46]. These tree-based algorithms demonstrate greater robustness against overfitting when trained on sparse datasets and provide inherent interpretability through feature importance scores, enabling researchers to isolate and investigate the contribution of specific chemical groups to defect passivation [45,46]. To provide a concise guide for algorithm selection, we summarize the comparative advantages and limitations of these methods, with a particular focus on data efficiency, in Table 2.

Beyond standalone models, integrated screening workflows have been developed to streamline discovery processes. For instance, the recently developed Co-PAS framework exemplifies this approach by employing a hierarchical screening strategy. It utilizes a Molecular Skeleton Classifier (MSC) for pre-screening and combines a Junction Tree Variational Autoencoder (JTVAE) with molecular descriptors to enhance the accuracy of photovoltaic conversion efficiency predictions [22]. This data-driven approach successfully narrowed down 250,000 candidates to a manageable list of high-potential organic additives, effectively bridging the gap between high-throughput screening and low-throughput experiments.

However, traditional screening is fundamentally limited to known samples within existing libraries [47]. To transcend this boundary, the field is witnessing a paradigm shift toward generative AI models in reverse molecular design [48]. Generative models such as Variational Autoencoders (VAEs) and Generative Adversarial Networks (GANs) function by mapping discrete molecular structures onto a continuous latent space [49]. The optimization algorithm can then navigate this manifold to propose entirely novel molecular structures, thereby achieving the desired properties of the optimized target [50,51].

## 4. Intelligent Process Optimization Strategies

### 4.1. The Challenge of High-Dimensional Parameter Spaces

Identifying an effective additive molecule represents only the initial stage of the material discovery process. The ultimate photovoltaic performance also hinges on precise processing conditions during perovskite film crystallization. These conditions constitute a vast, high-dimensional parameter space. This space is not limited to simple concentration metrics but encompasses the entire manufacturing protocol, including dynamic spin-coating kinetics and environmental synthesis constraints. Influenced by factors such as additive concentration, solvent mixing ratios (e.g., DMF:DMSO), annealing heat treatment curves, and the antisolvent addition time window [44]. ML methods have demonstrated strong capabilities in capturing nonlinear relationships during device optimization, such as identifying the dominant influence of annealing temperature and molecular chemical characteristics relative to simple concentration metrics [29]. Moreover, interpretable analyses such as SHAP reveal complex interactions between precursor solvent composition and cation ratios [52], while a fundamental understanding of solvent polarity remains crucial for regulating crystallization kinetics and defect passivation mechanisms [53].

Traditional optimization strategies rely on conventional trial-and-error methods. Due to the diverse and complex factors affecting equipment efficiency, these strategies are typically time-consuming, labor-intensive, and inefficient [54]. Traditional experimental methods typically analyze individual factors in isolation, failing to capture complex nonlinear interactions inherent in the full synthesis and manufacturing protocol, and potentially missing optimal solutions within multi-parameter design spaces [44]. As the number of variables increases, the experimental effort required to map this space grows exponentially. This is known as the curse of dimensionality [23,55]. This combinatorial complexity renders traditional brute-force methods intractable, necessitating the adoption of data-efficient sequential learning frameworks.

### 4.2. Active Learning and Bayesian Optimization Strategies

To navigate these high-dimensional parameter spaces with limited experimental budgets, the research paradigm is shifting from passive static analysis toward AL, with BO serving as the gold standard [56,57]. Unlike passive approaches, BO employs probabilistic surrogate models, typically Gaussian Processes (GP), to quantify prediction uncertainty alongside mean performance [58,59]. This capability is particularly critical for addressing the sparse dataset problem in perovskite research [60], as it allows the algorithm to strategically balance the exploitation of known high-performance regions with the exploration of uncertain, potentially high-reward formulations. Furthermore, recent methodologies have integrated coarse-grained estimation strategies to optimize these models, leveraging low-fidelity proxy signals to compensate for the scarcity of high-precision data [61,62].

**Figure 2:**
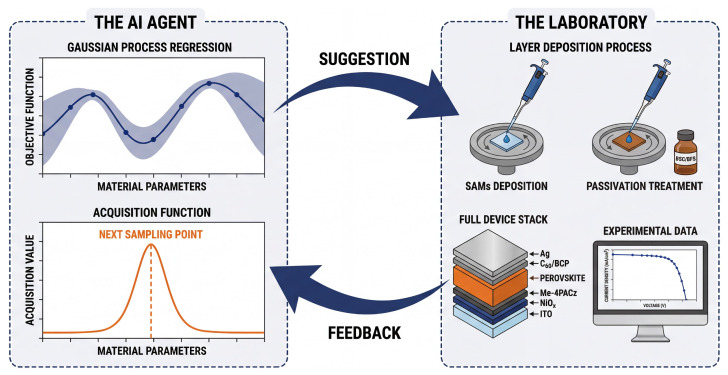
The BO closed-loop workflow for perovskite discovery. This cycle is driven by an AI agent (**left**), which uses a surrogate model, typically Gaussian process regression, to predict material properties and quantify uncertainty (shaded area). The objective function then balances exploration and exploitation to propose the next set of optimal material parameters. These suggestions further guide physical experiments in the lab (**right**), such as spin-coating preparation and characterization. Finally, newly measured data is fed back to update the model, thereby reducing uncertainty and optimizing subsequent predictions.

The decision on which experiment to perform next is determined by the acquisition function, which navigates the exploration-exploitation tradeoff by balancing sampling in areas with higher predicted performance (exploitation) against sampling in areas with higher uncertainty (exploration) [57]. Consequently, the decision to test a candidate is not solely based on high prediction confidence or reliability. Instead, candidates with high uncertainty are often deliberately prioritized if they offer significant information gain to refine the model boundaries. Through this iterative process (as shown in Figure 2), the system can rapidly converge to the optimal processing window [63].

Adaptive capabilities are particularly crucial for environmental perception processing, as environmental variables undergo dynamic changes. To address this challenge, a knowledge-constrained BO framework has been developed to maintain process stability under real-time conditions [16]. When confronted with a multidimensional space involving composition and processing conditions, researchers incorporated domain knowledge and prior data as probabilistic constraints into the acquisition function. The final results demonstrate that the system successfully identified stable candidates within a budget of fewer than 100 experiments, achieving a success rate over five times higher than that of traditional sampling strategies.

### 4.3. Solvent and Additive Synergistic Optimization

Identifying ideal additive molecules is merely the first step, as the effectiveness of these functional molecules strictly depends on their solvent environment [39]. Past studies have demonstrated that specific physicochemical descriptors of solvents, particularly the dielectric constant ($\varepsilon_{r}$) and Gutmann donor number ($D_{N}$), are key characteristics determining whether additives can effectively modulate crystallization kinetics [64]. Integrating this fundamental domain knowledge into the ML framework can significantly enhance the predictive capabilities of model [62].

This dependency stems from competitive coordination equilibrium discussed in Section 2.2. Since high-$D_{N}$ solvents (e.g., DMSO) form strong adducts with ${\mathrm{Pb}}^{2+}$, additives must compete for coordination sites or undergo ligand exchange to interact with the metal center [39]. Therefore, ML models must not treat additives in isolation. Instead, they should incorporate solvent descriptors to capture high-dimensional interactions with environmental variables [65], while also analyzing the nonlinear trade-offs between molecular properties of the additives [22]. By learning from high-dimensional formulation data, these models may be employed in the future to propose suitable solvent-additive ratios, thereby achieving critical equilibria. Specifically, achieving interactions strong enough to stabilize the intermediate phase and delay nucleation, while remaining sufficiently weak to permit controlled dissociation during annealing [66].

## 5. AI-Enabled Mechanistic Insights

### 5.1. Explainable AI for Rational Design

**Figure 3:**
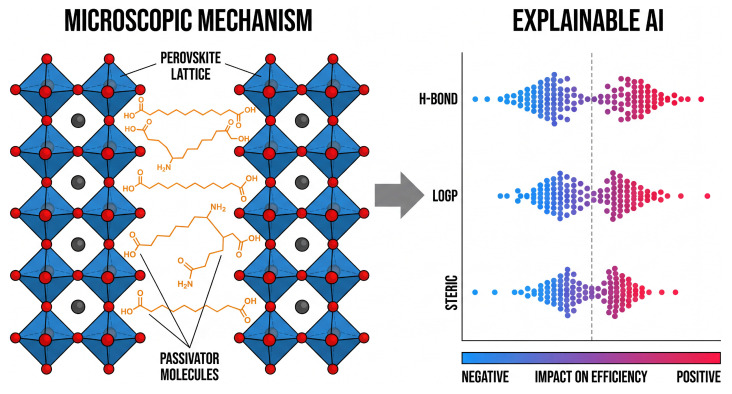
Integrating microscopic mechanisms with explainable AI. (**Left**): Schematic of perovskite grain boundaries, where passivating molecules (orange) anchor to lattice defects. (**Right**): Corresponding visualization provided by explainable AI, represented using SHAP swarm plots. This figure quantifies the impact of specific molecular features—such as hydrogen bonding, LogP values, and steric effects—on device performance, enabling rational design based on distinct chemical mechanisms. The vertical dashed line indicates a SHAP value of zero, separating negative (left) and positive (right) impacts.

Although deep learning models show promise in demonstrating predictive capabilities for screening potential additives, their inherent black-box nature often obscures underlying chemical logic, thereby limiting further understanding of material design. To address this trade-off between interpretability and accuracy, explainable AI methods, particularly SHAP (Shapley Additive Explanations), are increasingly being integrated into discovery workflows. By leveraging game theory to decompose prediction outputs into marginal contributions from individual features, researchers can now bridge the gap between high-dimensional data correlations and physical interpretations (Figure 3) [52,54].

To distinguish true mechanistic discovery from post hoc rationalization, we highlight the study by Ragni et al. [29]. Their SHAP analysis revealed the non-intuitive chemical insight that molecular flexibility as quantified by the number of rotatable bonds proved to be a more decisive predictor for surface passivation than electronic binding strength alone. This finding shifted the design paradigm from merely seeking strong Lewis bases to prioritizing structurally adaptive spacers capable of conformal coverage over grain boundaries, representing a crucial steric rule that was previously overshadowed by electronic considerations.

### 5.2. From Black-Box Screening to White-Box Kinetic Inference

Current AI-based research on perovskite additives is primarily dominated by black-box methods. These approaches have proven exceptionally effective in exploring the vast chemical space of potential additives, successfully identifying optimal concentrations that maximize film quality or device stability [63,67]. However, despite excelling at predicting effective methods, these data-driven models typically fail to explain why specific additives alter the crystallization process. They treat the formation process as an opaque mapping from initial points to final indicators, overlooking the underlying kinetic control equations that ultimately determine morphology.

To overcome this limitation, Scientific Machine Learning (SciML) offers a white-box alternative by embedding physics-driven dynamical laws into the learning process. Dahl et al. [68] demonstrated a pioneering example of this mechanism by developing a coupled spectroscopic-dynamical model to elucidate the formation process of two-dimensional perovskites. By constraining the neural network framework using a system of Ordinary Differential Equations (ODEs) describing the laws of mass action, they successfully deconvoluted competing formation pathways. They obtained precise rate constants from over 500,000 in situ absorption spectra.

Although Dahl’s work focused on colloidal systems, it provides a blueprint for the field of additives. Future research may adopt similar SciML frameworks to quantitatively extract how specific functional groups alter nucleation activation energy or diffusion-limited growth rates in films. Bridging high-throughput black-box screening with SciML-based kinetic inference may represent a key frontier for achieving ideal additive engineering.

### 5.3. Accelerating Molecular Dynamics via ML Potentials

Traditional first-principles methods, such as Density Functional Theory (DFT), are often limited in simulating atomic-scale systems due to their computational complexity scaling steeply (approximately cubically) with system size. Consequently, DFT is often confined to small systems (e.g., fewer than $10^{3}$ atoms) and short timescales (picosecond range), rendering it inadequate for realistically modeling grain boundary dynamics or passivation processes in polycrystalline perovskites. To overcome this limitation and bridge the spatiotemporal gap, Machine Learning Interatomic Potentials (MLIPs) have emerged as a powerful approach. Trained on DFT-based energies and forces, MLIPs can approximate Potential Energy Surfaces (PES) with accuracy close to first-principles calculations while offering computational costs comparable to classical force fields. This enables large-scale Molecular Dynamics (MD) simulations at the million-atom scale and nanosecond timescale [69,70].

Recent studies have applied MLIP to halide perovskite. For instance, researchers modeled a CsPbBr_3_ grain boundary containing approximately 6400 atoms and simulated ion migration at the nanosecond timescale. This revealed that halide (Br) diffusion preferentially occurs at grain boundaries, exhibiting significantly lower activation energy compared to the bulk phase. This demonstrates that the MLIP method can capture authentic grain boundary structural rearrangement and ion migration processes at device-relevant scales [71]. Similarly, in bulk halide perovskites such as CsPbI_3_, relevant studies have employed MLIP to simulate the diffusion of charged halide interstitial atoms and vacancies, thereby providing insights into defect dynamics beyond the capabilities of static DFT calculations [72].

Although MLIPs cannot fully replace ab initio methods, particularly when handling configurations outside extreme distributions or complex electronic effects [69], they represent a scalable and practical compromise. Thus, it can be stated that ML-based Molecular Dynamics (ML-MD) has become a viable approach for atomic-level modeling of ion migration and defect dynamics in perovskites under certain conditions [71,72].

Looking forward, extending MLIPs to additive engineering represents a complex but necessary frontier. To practically include additives, future training datasets would need to go beyond bulk structures to explicitly encompass “additive-surface” supercells that sample diverse adsorption configurations. We anticipate that the main risk in this expansion lies in chemical transferability: potentials trained on specific functional groups may struggle to generalize across the vast chemical space of additives without extensive retraining, and accurately capturing weak dispersion forces at the interface will remain a critical challenge.

## 6. Generative Design and Autonomous Agents

### 6.1. Inverse Design via Latent Space Navigation

Although high-throughput screening has successfully mined numerous highly effective materials and additives from commercial libraries, it remains constrained by the finite boundaries of the search space. A paradigm shift is underway in materials design toward AI-driven inverse design. Unlike discriminative models that map structures to properties, generative models such as VAEs enable efficient exploration of vast, unexplored chemical spaces by mapping discrete, high-dimensional chemical or structural data into continuous, low-dimensional latent spaces [49,73].

**Figure 4:**
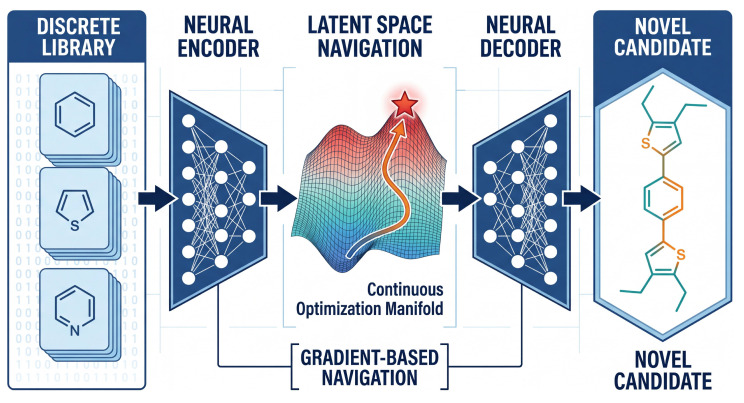
Schematic of generative inverse design via latent space navigation. Discrete molecular structures from existing databases (**left**) are mapped to a continuous low-dimensional latent space via an encoder. Within this differentiable manifold, optimization algorithms perform gradient-based navigation (orange trajectories) across the property landscape (colored surface) to identify the optimal solution (red star). A decoder then reconstructs these optimized latent vectors into novel candidates (**right**), molecules that may not exist in the original training dataset.

The core advantage of this compressed representation lies in its differentiability. Optimization algorithms can traverse this continuous manifold (as shown in Figure 4) along property gradients to find latent vectors that maximize or optimize specific objectives, such as thermodynamic stability, defect binding energy, tolerance factor, or other design metrics. These vectors are then decoded into entirely novel molecular or crystal structures that may not exist in any predefined library [49].

Although the direct application of generative models to small-molecule additives remains in its infancy, pioneering work on perovskite host materials and cation design demonstrates the immense potential of this approach. For instance, researchers have proposed a framework called the Evolutionary Variational Autoencoder for Perovskite Discovery (EVAPD) to generate novel perovskite stoichiometries, including dual A-site and B-site variants, while optimizing their formation energies and structural feasibility [73]. Building upon this foundation, subsequent research addressed the limitations of low-symmetry lattice reconstruction by introducing the Lattice-Constrained Material Generation Model (LCMGM). This architecture incorporates an auxiliary GAN to rigorously enforce geometric constraints, ensuring generated candidate materials conform to specific high-symmetry crystal systems such as cubic or tetragonal phases [49]. Additionally, a recent study reported the characteristic generation of organic A-site cations, which aims to regulate the dimension and stability of low-dimensional perovskites. This work demonstrates how generative molecular design can target additive or spacer-related structural and size parameters within perovskite systems [74].

These generative workflows are particularly advantageous for additive and interstitial engineering, as they enable simultaneous optimization of potentially conflicting properties. For instance, maximizing passivation efficiency while minimizing hygroscopicity or mismatch with the perovskite lattice. This capability makes it possible to explore the Pareto frontier of chemical space beyond existing compound libraries. Thus, a promising direction emerges when generative design is combined with performance predictors, physical constraints including tolerance factors and lattice compatibility, and downstream validation using DFT and experiments. This opens the possibility of discovering novel additives and passivators highly unlikely to emerge through traditional screening or enumeration methods [74,75].

### 6.2. Large Language Models as Cognitive Agents

LLMs are increasingly being deployed in materials research to address the explosive growth of published literature, extract domain knowledge, and assist hypothesis generation. For instance, the Perovskite-LLM system is a domain-specific LLM tailored for PSC research. To support this system, the authors constructed a comprehensive knowledge graph, termed Perovskite-KG, from 1517 research papers. This graph contains 23,789 entities and 22,272 relationships. Building upon this structured knowledge base, specialized LLMs were trained for question-answering and scientific reasoning, demonstrating significant superiority over general-purpose models in domain-specific tasks [24].

Furthermore, foundation models adapted for specific domains, such as LLaMat fine-tuned on materials science dataset, demonstrate superior performance in structure extraction, crystal generation, and information retrieval compared to general-purpose LLMs [76]. More broadly, integrating LLMs with techniques such as retrieval-enhanced generation, knowledge graphs, or domain-specific fine-tuning can significantly accelerate material discovery. These approaches facilitate critical tasks including crystal structure prediction, defect analysis, literature mining, and hypothesis synthesis [77].

Regarding experimentally validated discovery, Perovskite-R1 [25] recently identified non-obvious additives (e.g., 5-hydroxy-2-methylbenzoic acid) that were overlooked by experts. Experimental validation confirmed that these AI-proposed candidates improved device efficiency (from 18.30% to 18.63%) and stability, outperforming manually selected analogues. This serves as a proof-of-concept that LLMs can transcend human cognitive biases to generate valid chemical hypotheses, moving beyond passive knowledge retrieval.

In principle, such LLM-based tools can assist additive and passivator research by mining diverse reports, cross-referencing data on solvents, additives, defects, and stability, and proposing novel additive candidates or formulation schemes that may not exist in traditional chemical libraries. This capability effectively guides subsequent computational screening or experimental validation. However, significant challenges remain in practice, particularly ensuring factual accuracy of generated chemical proposals and minimizing fictional content [24]. Additional challenges include meaningfully linking natural language outputs to quantitative predictions and verifying the synthetic feasibility of generated molecules. Therefore, LLMs should currently be regarded as tools to assist hypothesis generation, serving as a complement to quantitative modeling or empirical validation rather than a replacement [77].

### 6.3. Closed-Loop Autonomous Discovery

**Figure 5:**
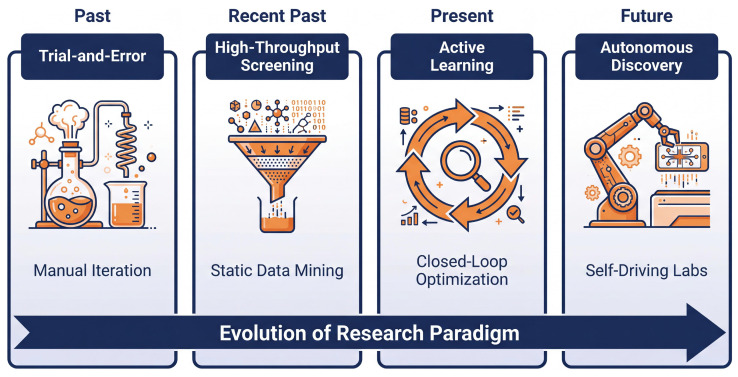
The evolution of research paradigms for perovskite additive engineering. This roadmap (indicated by the bottom arrow) illustrates the evolution of material discovery methods from trial-and-error approaches reliant on human intuition (Phase 1) to high-throughput screening (Phase 2), which utilizes static data mining to select candidate libraries. Active learning introduces dynamic closed-loop optimization (Phase 3) to navigate complex processing spaces (Section 4). The ultimate vision is the realization of SDL (Phase 4), where integrating AI agents (e.g., LLMs) with robotic platforms enables self-driving laboratories to accelerate innovation without human intervention (Section 6).

The convergence of generative design, reasoning agents, and robotic automation points toward the realization of Self-Driving Laboratories (SDLs). In these laboratories, AI algorithms are tightly integrated with automated preparation and characterization, thereby closing the Design-Make-Test-Analyze (DMTA) loop of experiments. This marks an evolution toward fully autonomous discovery (as illustrated in Figure 5). For perovskite materials, the robotic platform PASCAL has demonstrated this potential by automating precursor mixing, spin coating, annealing, thin-film formation, and in-line optical characterization. Through these capabilities, PASCAL enables high-throughput exploration of the composition and process parameter space for halide perovskite films more efficiently and consistently than manual methods [26].

More broadly, SDLs, such as those featured in the General Film Discovery Research lab, have successfully applied model-based optimization algorithms to explore complex parameter spaces involving composition, process, and environmental conditions in a closed-loop manner. This approach has enabled accelerated optimization for optoelectronics and material properties [61]. Extending these autonomous laboratory concepts to perovskite additive engineering will enable AI-generated candidate molecules to be synthesized, tested, and analyzed immediately. This integration significantly reduces the time lag between hypothesis generation and experimental feedback, making rapid iteration possible across the high-dimensional space encompassing additives, processing parameters, and compositions.

Nevertheless, fully autonomous additive discovery processes still face significant challenges. These include integrating reliable, chemically aware robots, particularly for synthesizing small-molecule additives, and ensuring robust characterization, such as evaluating film uniformity and performing defect or impurity analysis. Furthermore, combining experimental feedback with accurate predictive models for defect passivation, long-term stability, and environmental tolerance remains difficult. Therefore, while SDL represents a promising avenue for high-efficiency discovery in perovskite additive engineering, it should currently be viewed as an ideal direction rather than a mature methodology.

## 7. Challenges and Future Perspectives

Although AI-based additive engineering holds transformative potential, several key challenges must be addressed before its widespread adoption becomes a reality.

First is the data dilemma. Unlike fields such as computer vision that benefit from large standardized datasets, perovskite research suffers from severe data fragmentation and heterogeneity. Critical metadata, such as glove box humidity, additive purity, and thin-film coating conditions, is frequently omitted in literature, undermining reproducibility and limiting the generalization capabilities of ML models. Even when process information is included, a recent study demonstrates that low data reproducibility and heterogeneous process representations significantly constrain the predictive performance of ML models for PSCs [78]. Therefore, the community must prioritize establishing standardized reporting protocols and open-access repositories that adhere to FAIR principles (Findable, Accessible, Interoperable, Reusable). For instance, relevant efforts have already been made to aggregate extensive PSC data (e.g., >42,000 devices) for meta-analysis [41,79]. While standard random splitting remains prevalent in current literature, it fails to prevent data leakage arising from structural redundancy and batch correlations. Therefore, to ensure rigorous validation, we strongly recommend adopting “scaffold splitting” [22] for additive libraries and “group-based splitting” for device datasets rather than simple random sampling. Furthermore, to mitigate the reporting bias inherent in “champion” device efficiencies, future benchmarks should shift learning targets from peak PCE to statistical metrics (e.g., average PCE) or stability indices (e.g., $T_{80}$ lifetime) [5]. Specifically, we now highlight the work by Liu et al. [16] as a key example, where incorporating process stability constraints, rather than solely maximizing PCE, into the optimization loop significantly improved reproducibility. To consolidate such efforts, we advocate for shared “Community Benchmarks” that explicitly standardize critical metadata (e.g., purity, humidity), statistical targets (average PCE, $T_{80}$), and rigorous scaffold-splitting protocols to prevent data leakage.

Second, the opacity of complex AI models remains a significant hurdle. Deep learning and generative approaches often function as black boxes, capable of producing highly accurate and high-quality predictions yet lacking mechanisms for interpretability. For ideal additive design, models must adhere to fundamental physicochemical constraints. Otherwise, proposed candidates may be chemically unreasonable or synthetically infeasible. Future developments should emphasize physicochemically informed architectures and hybrid workflows that integrate AI suggestions with first-principles calculations and experimental validation.

Third, experimental bottlenecks persist. Even if AI can generate promising additive candidates or processing schemes within seconds, wet lab synthesis, film fabrication, and full device testing typically require days or longer. Integrating state-of-the-art domain-specific LLMs with SDL represents a promising approach to alleviate this issue. However, achieving this demands robust, chemically aware automation systems, reliable characterization methods, and seamless integration between AI-driven design and physical implementation [79]. Before such infrastructure becomes widespread, extensive autonomous discovery should be viewed as a long-term goal rather than a current reality.

Looking ahead, the most impactful advances may come from multimodal foundational models capable of synthesizing information across text, structured data, images, and representational data. Combined with SDLs, this could enable AI to propose, fabricate, test, and iterate perovskite additive formulations within a closed-loop cycle, significantly accelerating the discovery process. Realizing this vision will require concerted efforts across the community in data standardization, comprehensive experimental metadata sharing, and experimental workflow development.

## Figures and Tables

**Table 1:** Common molecular descriptors and their physical significance in perovskite additive screening.

Descriptor Type	Specific Metrics	Physical Meaning/Mechanism
Geometric & Structural	Molecular Weight, Rotatable Bonds	Quantify steric hindrance and conformational entropy [29]. Excessively large molecules may distort the crystal lattice, while highly flexible molecules better adapt to the topology of grain boundaries.
Electronic Properties	HBD/HBA (Hydrogen Bond Donor/Acceptor) Counts	Indicators used to characterize binding affinity [17,21] and charge transfer. The number of HBD/HBA sites is directly correlated with the number of defect-passivated anchor sites.
Solubility & Polarity	LogP (Partition Coeff.), TPSA	Characterize phase distribution and moisture resistance [21,35]. Determine whether additives are integrated into the matrix or segregate to the surface, and define the hydrophobic barrier.
Topological Fingerprints	MACCS Keys, ECFP4	The presence of functional groups. Mapping specific substructures to passivation effects enables ML models to learn structure-property relationships [22].

**Table 2:** Critical comparison of AI methodologies in perovskite additive engineering.

Methodology (Algorithms)	Data Efficiency	Primary Application	Key Strength/Limitation
Ensemble Learning (Random Forest, GBDT)	High (<100 points)	Screening from libraries	Pros: Robust on small data; interpretable. Cons: Poor extrapolation to new chemicals.
Deep Learning (GNN, DNN)	Low (Data hungry)	Feature Extraction	Pros: Captures complex topology/structure. Cons: High overfitting risk without pre-training.
Bayesian Optimization (Gaussian Processes)	Very High (Sequential)	Process Optimization	Pros: Best for small experimental budgets. Cons: Scales poorly with high dimensionality.
Generative Models (VAE, GAN)	Moderate (Latent space)	Inverse Design	Pros: Explores “Unknown Unknowns”. Cons: Hard to ensure synthetic feasibility.

## Data Availability

No new data were created or analyzed in this study. Data sharing is not applicable to this article.
